# Understanding parents’ decisions to seek care for children with serious bacterial infections: a qualitative study

**DOI:** 10.1136/bmjopen-2025-115270

**Published:** 2026-06-24

**Authors:** Evelien B van Kempen, Maarten Willemsen, Rianne Oostenbrink, Mirjam van Veen

**Affiliations:** 1 Department of General Pediatrics, Erasmus MC Sophia, Rotterdam, The Netherlands; 2 Department of Pediatrics, Juliana Children’s Hospital, Haga Hospital, The Hague, The Netherlands

**Keywords:** Parents, ACCIDENT & EMERGENCY MEDICINE, Paediatric A&E and ambulatory care, Paediatric infectious disease & immunisation, QUALITATIVE RESEARCH

## Abstract

**Abstract:**

**Objective:**

To explore which factor or combination of factors is perceived by parents as most distinctive in prompting them to seek medical care for children aged 0–5 years who were admitted to the hospital with fever due to a serious bacterial infection (SBI), in order to inform further development of an e-health tool.

**Design:**

A qualitative study was conducted using semi-structured interviews with a purposive sampling strategy. Interviews were conducted by a medical intern and supervised by a clinician researcher. Interviews were audio-recorded, transcribed verbatim and analysed using thematic analysis to identify key themes in parental decision-making.

**Setting:**

Paediatric ward of the Juliana Children’s Hospital in The Hague, an urban teaching hospital in the Netherlands, between April and June 2024.

**Participants:**

Parents of children aged 0–5 years admitted with a suspected SBI were eligible. Of the 17 parents approached, 15 agreed to participate in 13 interviews (ie, in two interviews both parents participated).

**Results:**

13 interviews with 15 parents revealed five themes. Parents sought medical attention when they perceived a shift in their child’s illness state, based on three themes: perceived abnormal illness presentation through either single alarming symptoms (altered consciousness, breathing difficulties, skin changes) or symptom combination, symptom recognition based on previous experiences or prolonged fever and respiratory complaints. Many parents demonstrated awareness of symptoms aligning with professional red flag indicators. The fourth and fifth themes, work constraints and limited general practitioner access, highlighted external barriers that sometimes delayed help-seeking.

**Conclusion:**

This study highlights key factors influencing parents’ decisions to seek medical care for febrile children with an SBI, emphasising the importance of both parental perceptions and clinical symptoms. The findings may inform parent-centred resources including an e-health tool, which support parents in assessing illness severity and deciding when to seek medical care.

**STRENGTHS AND LIMITATIONS OF THIS STUDY**

- Semi-structured interviews allowed parents to describe their experiences in depth while ensuring that key topics were addressed across interviews.
- Purposive sampling enabled inclusion of parents from diverse backgrounds.
- Interviews were conducted after the child’s diagnosis, which may have influenced parents’ recall and description of symptoms.
- The sample included some parents with previous hospital-care experience, which may limit transferability to parents with less healthcare experience.

## Introduction

 Febrile illnesses are one of the most frequent causes of paediatric visits to the emergency department (ED).^1^,^4^ While most cases are due to self-limiting viral infections, approximately 10%–15% of children are diagnosed with a serious bacterial infection (SBI) at the ED.^4 5^ Early identification of an SBI can be challenging, as the initial presentation can be difficult to distinguish from benign viral illnesses.^4 6 7^ Delayed recognition of clinical deterioration is one of the leading contributors to preventable deaths among hospitalised children.^8^ Consequently, clear discharge instructions and adequate safety netting are important to support parents monitoring their child at home.^9^,^16^ However, previous studies have highlighted that the information and instructions provided by physicians on discharge often fall short in meeting the knowledge and needs of parents.^9^,^1115^ Parents find it challenging to assess the severity of their child’s febrile illness, which may result in two opposing consequences: unnecessary visits to the ED due to misinterpreting symptoms or unclear safety netting advice and delayed help-seeking, potentially due to previous negative experiences.^9 10 16 18 19^ Both of these scenarios can increase the risk of adverse outcomes, including morbidity and mortality.

To address this gap, previous studies have underscored the need for clear, consistent and reliable information for parents about fever, including materials that can be consulted at home.^910 12^,^15 20^ Kuijpers *et al* recommended the development of a checklist based on signs and symptoms that parents can identify, which could support them in monitoring their child’s condition following discharge and improve communication with healthcare providers.^9^ In their qualitative study, parents primarily relied on their child’s usual behaviour and physical appearance as a reference point to interpret illness signs and symptoms and to assess severity. Parents may find it difficult to recognise specific clinical symptoms, like dyspnoea or dehydration, which are commonly used in physician instructions, especially when they had no prior experience with these symptoms.^9 21 22^ On the other hand, parents may detect subtle behavioural changes indicative of clinical deterioration, often before deviations in vital signs are apparent.^12 20 23 24^

Building on this work, we initiated a phased project with the ultimate aim of developing an e-health tool to support parents in assessing the severity of febrile illness in young children. The tool will use a structured parent-reported questionnaire, based on the checklist of parent-reported signs and symptoms developed by Kuijpers *et al*, to guide assessment (available at zib.evidencio.com and in online supplemental material 1). Because the tool is intended to support parents in recognising potentially serious illness, it is important to examine whether the questionnaire reflects the signs and symptoms that parents consider most relevant in the context of SBI. Although parental experiences of childhood fever have been studied, evidence remains limited on how parents recognise and interpret signs of severity in children with an SBI, and how these perceptions influence their decisions to seek medical care. We therefore conducted the present qualitative study among parents of children aged 0–5 years admitted to hospital with an SBI. Using the tool’s structured questionnaire as a reference during the interviews, we explored how parents perceived signs of serious illness and how these perceptions shaped care-seeking decisions. The primary aim of this study was therefore to explore which factor or combination of factors parents perceived as most distinctive in prompting them to seek medical care, thereby inform further development of the e-health tool. A secondary aim was to explore which factors, beyond the child’s health characteristics, parents experienced as facilitating or hindering their decision to seek medical care.

To guide our exploration, we applied the established ‘Access to healthcare model’, adopted from Levesque *et al* (online supplemental material 2: figure S1).^25^ In this model, access to healthcare is defined as “the opportunity to reach and obtain appropriate health care services in situations of perceived need for care”. Access is understood as the result of complex interactions between individual and household characteristics, the broader social and physical environment and the organisation and functioning of health systems and providers. This framework allows for a comprehensive understanding of the various factors influencing healthcare-seeking behaviour, offering a structured lens to interpret the elements that guide parents’ decisions to seek medical care for their children.

## Methods

### Study design

We conducted a qualitative study underpinned by a content analysis methodological orientation, using semi-structured interviews with parents of children aged 0–5 years who were admitted to the hospital with fever due to an SBI. The reporting of this study was guided by the Consolidated Criteria for Reporting Qualitative Research (COREQ) guidelines.

### Recruitment

Participants were recruited at the Juliana Children’s Hospital, a large teaching hospital in The Hague, the third-largest city of the Netherlands. The hospital serves a diverse, urban population and its catchment area includes children from varied socio-economic and ethnic backgrounds. Participants were eligible if they had a child who: (1) was aged between 0 and 5 years, (2) was admitted to the paediatric ward, (3) had a reported fever (>38°C) within 24 hours prior to admission or measured at presentation and (4) was considered to have a fever due to an SBI, as determined by the treating clinician. The sole exclusion criterion was a language barrier, defined as an inability to communicate in Dutch at B1 level. Purposive sampling with maximum variation strategy was used to capture a diverse range of perspectives in terms of parental characteristics (number of children, gender, caregiving experience, socioeconomic status, educational level, cultural background and ethnicity). During morning ward rounds, the treating physician introduced the study to eligible parents. Next, a member of the research team (MW) provided further information. If parents agreed to participate, informed consent was obtained and participants completed a sociodemographic form (online supplemental material 3). The semi-structured interviews were conducted between April and June 2024.

### Data collection and analysis

A topic guide was developed based on the structured questionnaire, previous studies, existing literature and the ‘Access to healthcare model’ (online supplemental material 4). The questions were reviewed by paediatric experts (MvV, RO) and based on their feedback revisions were made. Parents were asked to recall the days leading up to the admission and to reflect on their responses to the questionnaire day by day. Interviews also explored decision-making moments, focusing on what influenced their choice to seek or delay care.

Interviews were conducted by MW and EBvK in Dutch, and lasted between 20 and 45 min. They took place in the child’s private hospital room to ensure comfort and allow parents to remain with their child. No repeat interviews were carried out. Semi-structured interviews were audio-recorded, after permission had been obtained. Field notes were taken immediately afterwards, including reflections on the interview. The interviews were transcribed ad verbatim in Dutch by MW, including non-verbal information. The transcripts were cross-checked by EBvK against the audio recordings. Transcripts were not returned to participants for feedback or correction due to time and resource constraints. After 10 semi-structured interviews, a preliminary analysis was performed to evaluate data saturation, which was reached after 13 interviews. Thematic analysis according to Braun and Clarke was used for analysis.^26^ EBvK and MW independently read the transcripts and both identified codes inductively. ATLAS.ti (V.24.1.1) was used. After coding five transcripts, codes were compared and refined until consensus was reached. The codes were grouped into subcategories and subthemes were identified to subsequently create overarching themes with the research group to achieve triangulation. For publication, selected illustrative quotes were translated into English by the research team, with attention to preserving participants’ intended meaning.

### Patient and public involvement

Parents were actively involved in the development of the questionnaire and e-health tool currently under development. The parents participating in the present qualitative study were separate from those involved in this earlier work and did not use the e-health tool during the study. The present study further informed the tool and its underlying questionnaire by exploring parents’ experiences of recognising signs of an SBI and seeking medical care. Parents and/or the public were not involved in the recruitment, conduct, analysis or dissemination plans of this study.

### Reflexivity

#### Reflections on the researchers themselves

MW is a seventh-year medical student conducting this study as part of his research project. He was supervised by EBvK, a medical doctor with an MSc in International Health, currently a third-year paediatric resident and PhD candidate. This study is part of her PhD project. EBvK has received formal education in qualitative research and has experience in qualitative research across various settings, including Nepal, Sweden, Sri Lanka and the Netherlands. This supervision allowed MW to receive guidance on both the methodological and practical aspects of qualitative research. To minimise her influence as a clinician, EBvK avoided wearing a white coat, adopted a neutral posture, encouraged parent empowerment and let MW take the lead in the interviews.

#### Reflections on research relationship

No relationship with the participants was established prior to the research commencement. The occupations of MW and EBvK were known to the participants. The aim to develop an e-health tool for parents of febrile children was shared with the participants. MW and EBvK emphasised the parents’ role as expert and importance of their insights. They showed genuine interest in their experiences. Trust was created and equality emphasised.

## Results

### Participant characteristics

A total of 13 interviews were conducted with 15 parents, including 10 mothers and five fathers. In two interviews, both parents participated (table 1). Two eligible participants did not participate due to lack of time and tiredness. Participants reflected a diverse range of professional backgrounds (table 2). The average age of the children was 2.2 years. Additional demographic characteristics are presented in tables1  2.

**Table 1:** Overview of interviews, participants and diagnosis at admission

Interview	Participant number	Relationship to child	Diagnosis
1	1	Mother	Respiratory infection
2	2	Father	Bone/Joint or soft tissue infection
3	3	Mother	Respiratory infection
4	4	Mother	Urinary tract infection
5	5	Father	Urinary tract infection
6	6,7	Mother (6) Father (7)	Respiratory infection
7	8	Mother	Urinary tract infection
8	9, 10	Father (9) Mother (10)	Respiratory infection
9	11	Mother	Respiratory infection
10	12	Mother	Urinary tract infection
11	13	Mother	Bone/Joint or soft tissue infection
12	14	Father	Respiratory infection
13	15	Mother	Central nervous system infection

**Table 2:** Aggregated participant and admitted child characteristics

Characteristic	Number
Participants	15
Relationship to child	
Mother	10
Father	5
Occupational category	
Healthcare	5
Administration	2
Education	2
ICT	1
Construction	1
Sales	1
Unemployed	1
Unknown	2
Admitted children	13
Diagnosis of admitted child	
Pneumonia	6
Pyelonephritis	4
Osteomyelitis	1
Meningitis	1
Preseptal cellulitis	1
Age admitted child, range in years	0–5
Number of children in family, range	1–4
Admitted child’s position among siblings	
Oldest or only child	9
Other	4

ICT, Information and Communication Technology.

### Overview of themes

Five main themes emerged from the data. The first three themes related to the primary aim and reflected the factors parents perceived as most distinctive in prompting them to seek medical care. This is captured by the overarching theme of perceived abnormal illness.

1. Perceived abnormal illness presentation: from the parents’ perspective, abnormal presentation of illness, based on either a severe disease characteristic prompting direct contact or a combination of non-severe disease characteristics, especially fever and dyspnoea.
2. Symptom recognition based on past medical experiences: recognition of symptoms from the past based on the medical history of the patient and family.
3. Prolonged illness duration: from the parents’ perception, an excessively long illness duration, particularly regarding fever or respiratory problems.

Two themes were identified in relation to the secondary aim, reflecting factors beyond the child’s health characteristics that parents experienced as facilitating or hindering their decision to seek medical care.

1. Work-related delays: delay in seeking medical care due to parental work commitments.
2. Absence of own general practitioner (GP), leading to earlier or delayed medical consultations.

**Figure 1:**
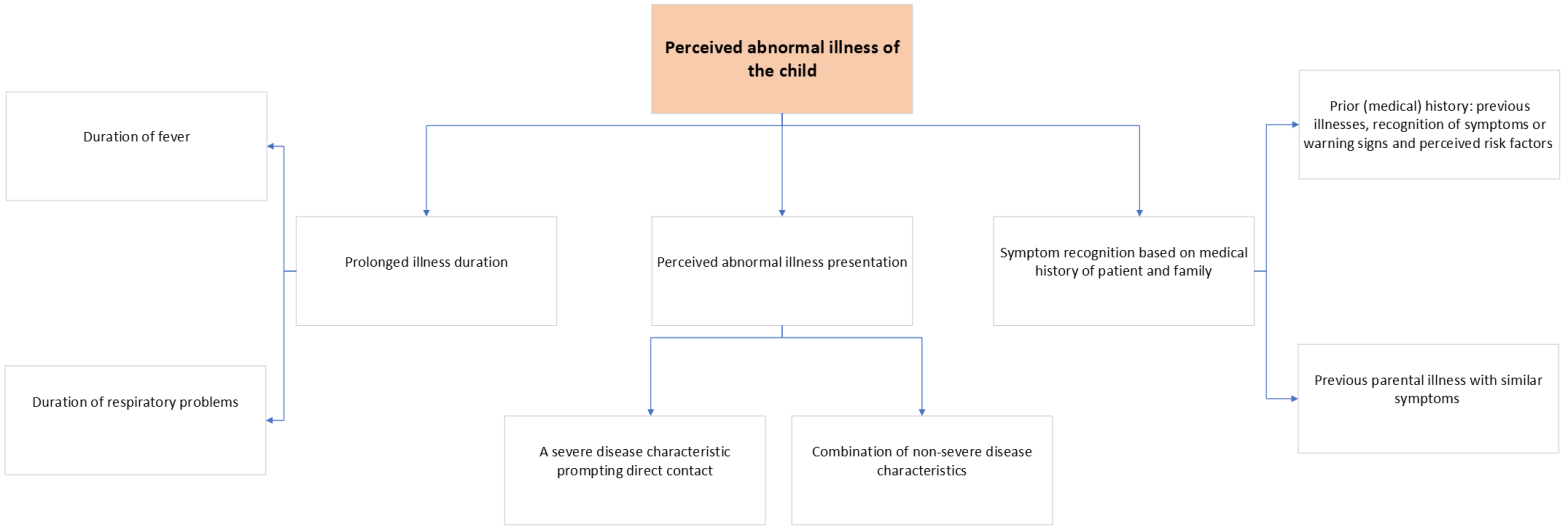
Thematic map showing the main themes integrated into the ‘Access to healthcare’ model, adapted from Levesque *et al*.^25^ The framework was adapted to reflect parental care-seeking for children with serious bacterial infection. Licensed under CC BY 2.0.

A thematic map was created to illustrate the themes and subthemes, structured according to the ‘Access to healthcare model’ (figure 1). Online supplemental material 5: table S1 provides quotes that exemplify each theme.

### Factors or combination of factors that parents found most distinctive in seeking medical care

**Figure 2:**
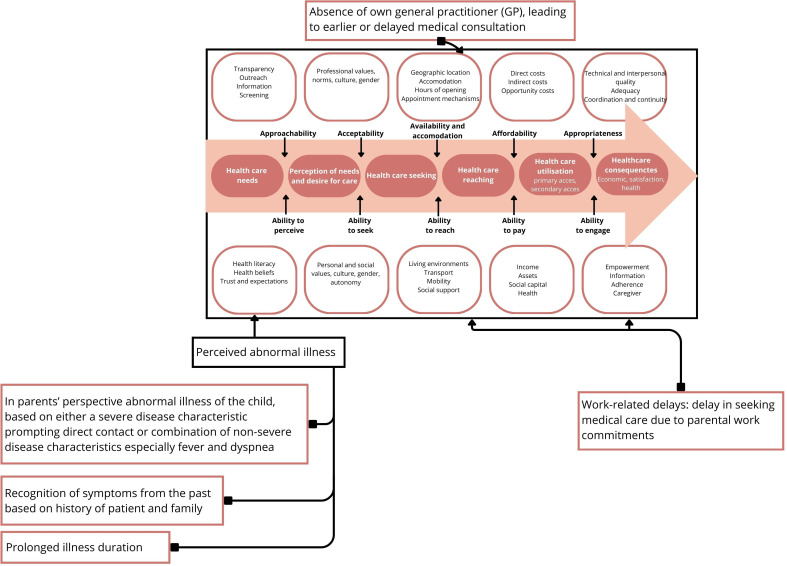
Parental perceptions of key factors prompting medical care-seeking for young children hospitalised with fever due to suspected serious bacterial infection (SBI). The figure shows the themes that parents identified as most distinctive in prompting them to seek medical care for children aged 0–5 years who were admitted to hospital with fever suspected to be caused by an SBI.

Initially, all parents perceived their child’s illness as typical for a febrile episode. However, at a certain point, they recognised a change in their child’s condition, which prompted them to perceive the illness as ‘abnormal’ and seek medical consultation. The factors contributing to this shift in perception are categorised into three themes, as outlined in figure 2.

### Theme 1. From the parents’ perspective, abnormal illness of the child, based on either a severe disease characteristic prompting direct contact or combination of non-severe disease characteristics, particularly fever and dyspnoea

Some parents observed a single, severe factor that triggered immediate medical consultation, while others were prompted by a combination of multiple individually non-alarming factors.

### Due to severe factor(s)

Parents described an altered level of consciousness, skin changes, severe respiratory problems and discomfort/suffering as individual red flags that directly prompted them to seek medical care. They found an altered level of consciousness—including symptoms such as reduced awareness, difficulty interacting and/or lack of engagement with their surroundings—the most alarming sign. One parent recounted that after her child’s afternoon nap,

she almost couldn’t wake him up” and “thought it might be serious enough to call an ambulance (P3).

Another explained

he would lose consciousness because he was simply exhausted. It seemed every time like he was about to faint and would not wake up again (P9). A third parent recalled,

he would lose consciousness every time, but when he was awake, he didn’t fully realize it. He would be eating a cracker that would fly out of his mouth and then ask why the wall is pink. That worried me greatly (P15).

Changes in skin colour and appearance were also considered potential indicators of a serious disease. Parents described various types of skin changes, most frequently pale skin and bluish discolouration, which often occurred together and triggered immediate contact with a physician. One parent recalled,

when we picked her up, I noticed that she was completely cold, turned extremely pale, and had blue lips. I thought that this situation was highly concerning and that we should go to a doctor immediately. I was at the point of either jumping into the car or calling emergency services, because I thought she was going to die (P4).

In some cases, the presence of petechiae (confirmed by clinician) led to urgent medical consultation, often influenced by the parent’s medical background or prior experience shared by relatives. As one parent described,

she had spots on her cheeks, which is a rash indicative of sepsis. I recognized it because I work in microbiology (P8).

Several parents described being alarmed by changes in their child’s breathing, including extreme coughing, use of accessory breathing muscles, chest indrawings and shortness of breath that did not improve with medication. In most cases, symptoms gradually worsened, eventually reaching a point at which parents sought urgent care. One parent shared,

he had a bit of a cold, that’s normal, that happens frequently. Then at one point I saw, while he took his afternoon nap, that his chest contracted a little and his breathing became heavier. Then we contacted the doctor (P11).

Some parents observed behavioural changes that led them to believe the child was suffering or in discomfort. One parent noted,

she had a high fever and was crying non-stop. She was very restless and not herself. You felt that she was suffering (P5)

and another added,

she was squealing… overstretching… not in a good mood. This is why we contacted a doctor (P4).

### Due to a combination of non-severe factors

In contrast, some parents were prompted to seek medical attention by the combination of multiple, individually non-severe symptoms. Specifically, the combination of fever and dyspnoea was cited as a reason for contacting a doctor in two interviews. One parent explained:

a fever in itself is not that bad, because with many viruses she has a fever and she also has shortness of breath with every little cold. So separately it is not that bad, but because it was a combination of factors we contacted the GP (P6).

### Theme 2. Recognition of symptoms from the past, based on the medical history of patient and family

Parents’ previous experiences shaped their interpretation of current symptoms, helping them anticipate deterioration. Two subthemes were identified: (1) the child’s medical history and (2) previous parental experience with similar symptoms.

### Child’s (medical) history: previous illnesses, recognition of symptoms or warning signs and perceived risk factors

#### Previous illnesses and recognition of symptoms or warning signs

A history of respiratory problems made parents more alert to subtle warning signs, such as retractions, nasal flaring or the use of accessory breathing muscles quicker. One parent described:

both her sister and she have been regularly hospitalised due to breathing difficulties that required oxygen. Retractions in her neck and ribs, the abdomen having to work harder, and nasal flaring are all things we know to watch for (P13).

Others identified a recurring pattern linked to a previous serious respiratory illness that heightened their concern:

a year ago, he had the RSV virus for which he was hospitalised. We initially thought that was the case again and therefore we contacted the GP (P1).

Past episodes of an SBI also heightened parents’ vigilance. As one mother explained:

my daughter previously had meningitis. Now I saw her not playing again, with a high fever, red cheeks, pallor, vomiting, and chills. I recognised these symptoms from earlier and thought: this won’t happen to me a second time. I didn’t want to contact the doctor too late, so I returned very quickly (P8).

#### Patient characteristics perceived as risk factor or rapid disease progression

Parents also cited perceived risk factors unrelated to the presenting symptoms, such as prematurity or a prior experience with rapid disease progression, as reasons for earlier consultation. One mother noted:

with her, it is very typical for things to escalate quickly. The shivering, the discomfort, and the drop in temperature have all happened before, followed by a rapid spike to 40.5 degrees. I thought: you are very cold now, but you will definitely have a high fever soon. That’s when I sought medical advice (P4).

### Previous parental illness with similar symptoms

In a few cases, parents linked their child’s symptoms to illnesses they had personally experienced or observed closely. For example, one father explained:

In the past, my wife had the dengue virus, which also caused a high fever. She became very ill from it. We immediately thought: could this be the same thing? (P5).

### Theme 3. In parents’ perception, an excessively long illness duration, especially of fever or respiratory problems

Parents had clear ideas regarding the typical duration of fever and respiratory complaints. When these symptoms persisted beyond their usual timeframe, it prompted consultation. Fever lasting 3–5 days was considered typical. As one parent noted,

fever usually peaks after a few days, but this time it persisted, so there was something wrong (P3).

Similarly, several parents mentioned that prolonged respiratory complaints, without signs of severe breathing difficulties, led them seek care if there was no improvement after 3–4 days. One parent described:

she was having difficulty breathing. Initially, it wasn’t getting better, but if it’s less than 3 days, you don’t really worry about it. Now it lasted longer than 3 days, so I thought, let’s go to the doctor (P7).

### Factors that parents found most facilitating or hindering in their decision to seek medical care, aside from the health characteristics of their child

Parents’ decisions to seek medical care were influenced by external factors, including work-related constraints and healthcare availability.

### Theme 4. Delay in seeking medical care due to parental work commitments

In two interviews, work obligations delayed parental healthcare-seeking despite concern about the child’s symptoms. One mother, whose child had worsening cough, breathing difficulties and fever, explained that she had already decided on Sunday that “things were not going well” but postponed contacting the GP on Monday because she had a midwife shift to work. Without it, she believed she “would have already gone to the GP on Monday”, but instead waited until Tuesday, yet “it wasn’t getting better… it was actually only getting worse” (P3). Similarly, a father described how his daughter, recently discharged from hospital, fell ill again after his wife had undergone surgery. Concerned about the repeated absences from work, he delayed seeking care, hoping she could manage with prolonged use of pain medication as he feared that further time off would create additional work complications. He shared, “I have been frequently absent and had to request a lot of leave due to my wife and daughter’s illnesses… Without my work concerns, I would have sought contact earlier” (P5).

### Theme 5. Absence of own general practitioner, leading to earlier or delayed medical consultation

In two interviews, parents explained that the GP’s involvement and familiarity with their child’s medical history influenced the timing of contact. One parent shared that the absence of their usual GP delayed seeking medical care despite growing concerns. As they recounted,

we thought on Saturday or Sunday that he needed to be seen, but it was Easter weekend, and he wasn’t so severely ill that he needed to be seen immediately. On Tuesday, the GP who had previously seen him was working, so we thought it would be most practical to consult him then (P2).

Conversely, another parent described how the approaching weekend prompted them to seek medical advice earlier than they might have based solely on the symptoms.

It is always concerning when she is short of breath. We’ve often had to call the after-hours clinic. You are always uncertain because they are not familiar with her. This time, she had a lot of trouble in the morning, so we thought it was best to go immediately, otherwise, she would have to go to the after-hours clinic instead of seeing her own physician (P6).

## Discussion

In this qualitative study of parents of children aged 0–5 years hospitalised with a febrile illness due to an SBI, parents generally sought help when they perceived a shift from a normal illness course to an abnormal or concerning state. This perceived abnormal illness was based on a single severe symptom, a combination of specific symptoms, their duration and prior parental experiences. Importantly, external factors like work obligations also influenced the process. Some delays were not caused by an inability to recognise the severity of the illness, but due to perceived barriers to accessing timely care. These findings align with the ‘Access to healthcare framework’, which emphasises that health-seeking behaviour is influenced by the interaction of individual, social and systemic factors.

### Comparison with literature

Based on previous research, we anticipated that parents would focus more on general behavioural changes than on specific clinical symptoms.^9 21 22^ However, our findings revealed that parental evaluation was more comprehensive, with parents also considering clinical symptoms. Particularly alarming to them were altered consciousness, changes in skin colour, breathing difficulties and observable suffering or discomfort. Importantly, these factors closely align with the ‘red flag’ symptoms in clinical guidelines like the National Institute for Health and Care Excellence (NICE) traffic light system.^3 27^ Our findings suggest that parents, especially those who mentioned previous experience, are (more) aware of clinical warning signs of serious illness, surpassing the recognition observed in earlier studies.^9 21 22 28^ Our findings align with previous qualitative studies exploring parental recognition of serious illness and childhood fever. Van den Bruel *et al*, in a study of 18 children hospitalised with a serious infection, found that parents identified drowsiness, irritability and different crying as key signs, along with their impression that illness was different.^6^ Similarly, Sahm *et al* conducted semi-structured interviews with 23 parents of children aged ≤5 years and found that petechiae and neck stiffness prompted parents to seek emergency care.^13^ The influence of previous parental experience on recognition and reporting is also highlighted in previous studies.^6 9 10 14 20 29^ These findings have implications for safety-netting advice. When explaining discharge instructions or tools such as the traffic light system, clinicians should consider parents’ prior experience and assess how they understand clinical warning signs. In addition, symptom duration, particularly the duration of fever and breathing difficulties, played a role in parents’ decision-making, supporting earlier observations.^610 29^,^31^ Safety-netting advice should therefore address which symptoms to monitor and how symptom persistence or progression should guide decisions to seek medical care.

While parents were often able to recognise concerning signs and symptoms, our findings suggest that access to healthcare could also be delayed by other barriers, like occupational obligations or limited availability of their own GP. There is a lack of studies exploring how similar challenges influence parental care-seeking in high-resource settings and the impact on the child’s outcome, although some have touched on this indirectly. In that case, the role of parental knowledge, beliefs and attitudes, social circumstances, barriers to primary care, presentation during weekend or evening shifts and conflicting information are mentioned.^9 10 14 16 18^

In this line, safety-netting advice and e-health tools should align with how parents interpret symptoms and make decisions. Previous studies have emphasised the need for clear and consistent information, including what symptoms to look for, home management and when and where to seek help.^10 13 15 17^ While national guidelines for the management of paediatric fever exist, their translation into accessible information remains needed.^10 13 14 32^ Our findings highlight that parents need clarity on which symptoms are cause for concern, tailored to both experienced and less-experienced parents, as well as clear guidance on when and how to seek help.

### Strengths and limitations

A key strength of this study is its emphasis on the signs that parents themselves identify as most distinctive and which trigger them to seek medical care for their child with an SBI. This perspective remains underexplored in the existing literature. Moreover, purposive sampling allowed for inclusion of parents with diverse backgrounds, broadening the scope of insights. Efforts to minimise social desirability bias through non-judgemental interviewing in a familiar and low-key setting further strengthened data quality. Furthermore, reflexivity was maintained throughout the research process to enhance the confirmability of the findings by critically considering the researchers’ own assumptions and positions. Field notes were used during interpretation and coding. Taken together, these methodological strengths contribute to the overall trustworthiness and credibility of the study.

Several limitations should be acknowledged. First, interviews were conducted after parents had already spoken with healthcare professionals and received a diagnosis for their child. This prior contact may have influenced how they perceived and described their child’s disease characteristics, potentially increasing their awareness of clinical signs typically emphasised by physicians. Second, the sample included several parents with previous experience navigating (in-)hospital care, which allowed us to identify signs they considered distinctive for SBI. However, prior experience was not systematically collected as a participant characteristic, so we could not determine how many parents had such experience. This may limit transferability to parents with less experience of navigating healthcare and should be considered when interpreting the role of prior experience in symptom awareness. Third, in some cases, a short delay between admission and the interview (due to weekends or holidays) may have affected recall of characteristics and decision-making process. However, this was mitigated by the structured interview approach, which included probing and a day-by-day reconstruction using the questionnaire, facilitating detailed and reflective accounts. Fourth, as interviews were conducted in Dutch and quotes were translated into English for publication, some nuance may have been affected during translation, although translations were checked by the research team to preserve participants’ intended meaning. Lastly, participants did not provide feedback on the findings due to time and resource constraints. Instead, credibility was enhanced through researcher triangulation, iterative analysis and regular team discussions to ensure that interpretations remained grounded in the data.

### Implications for future research and clinical practice

This study enhanced our understanding of how parents of children with an SBI perceive symptoms and decide when to seek care. These insights are important for developing healthcare interventions informed by parental experience, including the e-health tool developed within our phased project.^32^ Our findings suggest that parent-reported signs and symptoms should be considered alongside established clinical ‘red flag’ symptoms, such as those outlined in the NICE traffic light system. Because this study focused on children admitted to hospital with an SBI, the findings reflect a specific and more severe subpopulation of febrile children rather than the broader population of febrile children presenting to the ED. Future research should therefore examine how signs of severity identified in this study relate to clinical outcomes in broader and more diverse populations of febrile children.

These findings should be interpreted in light of the characteristics of the participating parents. Several parents had healthcare-related occupations or previous experience with serious illness or hospital care, which may have influenced their familiarity with alarming signs, their ability to describe symptoms and their threshold for seeking medical care. Most participants were mothers, although fathers also participated, and differences in caregiving roles may have influenced which illness signs were observed and emphasised during the interviews. Improving outcomes for febrile children requires attention to symptom recognition and to the non-medical barriers that shape when and how parents seek care.

Future research should involve non-Dutch-speaking populations and migrant populations, who may experience lower health literacy and greater barriers to accessing care. Understanding the barriers and needs of these groups is essential to ensure equitable implementation of interventions and access to care.

## Conclusion

This qualitative study offers insights into how parents of young children hospitalised with fever due to an SBI recognise illness severity and decide to seek medical care. Parents typically sought medical help when they perceived a change in their child’s condition based on single alarming symptoms, symptom combinations, previous experiences or prolonged fever and respiratory complaints. Many parents in our study, especially those with experience, recognised clinical warning signs aligning with ‘red flag’ indicators. However, when parents identified concerning symptoms, other barriers like work obligations and limited own GP availability could delay help-seeking. These findings may inform parent-centred resources that address clinical and behavioural symptoms as well as non-clinical barriers, supporting parents in assessing illness severity and deciding when to seek medical care.

## Supplementary material

10.1136/bmjopen-2025-115270 online supplemental file 1

## Data Availability

No quantitative datasets were generated or analysed for this study. The qualitative interview transcripts are not publicly available because they contain potentially identifiable information and their disclosure could compromise participant privacy and confidentiality. All data relevant to the study are included in the article or uploaded as supplementary information.
