# Supplementary material for: Understanding parents’ decisions to seek care for children with serious bacterial infections: a qualitative study
Source: BMJ Open. 2026 Jun 24;16(6):e115270. doi: 10.1136/bmjopen-2025-115270 (PMC13296029; doi:10.1136/bmjopen-2025-115270)
Supplement: online supplemental file 1 [file bmjopen-16-6-s001.pdf]

Supplementary materials

Supplementary material 1: Structured questionnaire employed in parental e-health tool Page 2-3
Supplementary material 2: Figure S1: Access to healthcare model Page 4
Supplementary material 3: Participant information and sociodemographic data form Page 5-8
Supplementary material 4: Topic guide (Dutch and English) Page 9-14
Supplementary material 5: Table S1: overview themes with illustrative quotes Page 15-16

Supplementary material 1: Structured questionnaire employed in parental e-health tool

This questionnaire's designed to help you assess how sick your child is.
The questions cover a range of topics. For example, how your child is drinking, urinating or playing.
Select the answer that best applies to your child at this moment
Once you've answered all the questions, you'll receive a summary for an overview of how ill your child is.

1. **How much is your child playing?**
   As much as normal
   Less than normal
   Not at all

2. **How active is your child?**
   As active as normal
   Less active or listless
   Not active or primarily lying down

3. **How well has your child been eating in the past 24 hours?**
   Like normal
   Not as good as normal (Is taking a few bites of a meal or snack)
   Has (nearly) stopped eating (pop-up: Is not eating or is only taking a few bites 1-2 times per day)

4. **How has your child been drinking in the past 24 hours?**
   Like normal
   Not as good as normal
   Is not drinking or only drinking a sip

5. **How's your child's urination?**
   My child's urinating as much as normal
   My child's urinating less than normal (pop-up: Fewer fully wet nappies than normal or just 1 full nappy per day)
   My child has (nearly) stopped urinating (pop-up: My child has not urinated for a day)

6. **How's your child sleeping?**
   My child's sleeping as much as normal
   My child's sleeping more, or more restlessly, than normal (pop-up: Your child cannot fall asleep properly and/or wakes up more often than usual)
   My child wants to sleep all day

7. **How alert is your child?**
   My child is as alert as usual
   My child's reacting sluggishly or only when I clearly address him or her
   My child does not respond to me or wakes up with great difficulty
   My child is confused (pop-up: For example, your child does not know where he or she is, is seeing things that are not there, does not know who you are, or is reacting strangely and differently than usual)

8. **How's your child's mood?**
   His/her mood is the same as usual
   My child's crying more easily than normal or seems in a bad mood (pop-up: When children are not feeling well, they're sometimes more quickly out of sorts. Your child may not be in the best of moods or may have less patience than usual. The latter can manifest as your child

crying more easily or being more irritable than normal)
My child's irritable, restless or crying all the time (pop-up: Your child's very angry, does not know what to do with him/herself and/or will not stop crying. Your child may be difficult to comfort and the crying may sound more plaintive than you're used to)

9. **How's your child talking/babbling?**
   Talking/babbling as much as normal
   Talks/babbles less and is quieter than normal
   No longer talking and/or babbling

10. **How well is your child establishing contact with you?**
    My child establishes as much (spontaneous) contact with me or more than usual (pop-up: My child seeks me out, looks at me, calls for me and/or smiles at me)
    My child's establishing less (spontaneous) contact with me than normal (pop-up: my child's responding to me when I go to him/her, but continues lying down or sitting and does not call for or seek me out)
    My child is establishing (almost) no contact (pop-up: my child's not responding to me)

11. **How does your child look?**
    My child looks like he or she normally does
    My child does not look like he or she normally does (pop-up: For example: red cheeks, slightly paler skin, a sweaty or sleepy look in the eyes)
    My child looks very pale or grey

12. **What is your child's temperature now?**
    There is no fever.
    Fever above 38°C.
    High fever, above 39°C

13. **How's your child responding to paracetamol?**
    My child has not had any paracetamol
    My child's feeling better after taking paracetamol.
    The paracetamol isn't helping

14. **Are you concerned about how sick your child is?**
    No, this is how my child acts when sick.
    Yes, I'm concerned because I want to know the cause/why my child is sick (pop-up:
    You may be concerned, for example, because:
    - You don't know if your child is seriously ill
    - You're worried that your child has a serious illness
    - You're worried because you do not quite understand why your child's ill)

    Yes, I'm worried because my child is not sick like he or she usually is (pop-up:
    Your child is behaving differently from the other times he/she has been ill. You feel like something's off or not quite right)

This page displays a summary of the answers you provided to the questionnaire. The coloured answer applies to your child. See the bottom of the page for the results.
The questionnaire will be completed again at a later time.
The summary indicates whether the number of your child's green, amber, or red symptoms has changed. More green symptoms are consistent with recovery. More red or amber symptoms may indicate that your child's condition is worsening and is a reason to contact your doctor.

Supplementary material 2: Figure S1: Access to healthcare model, adapted from Levesque et al

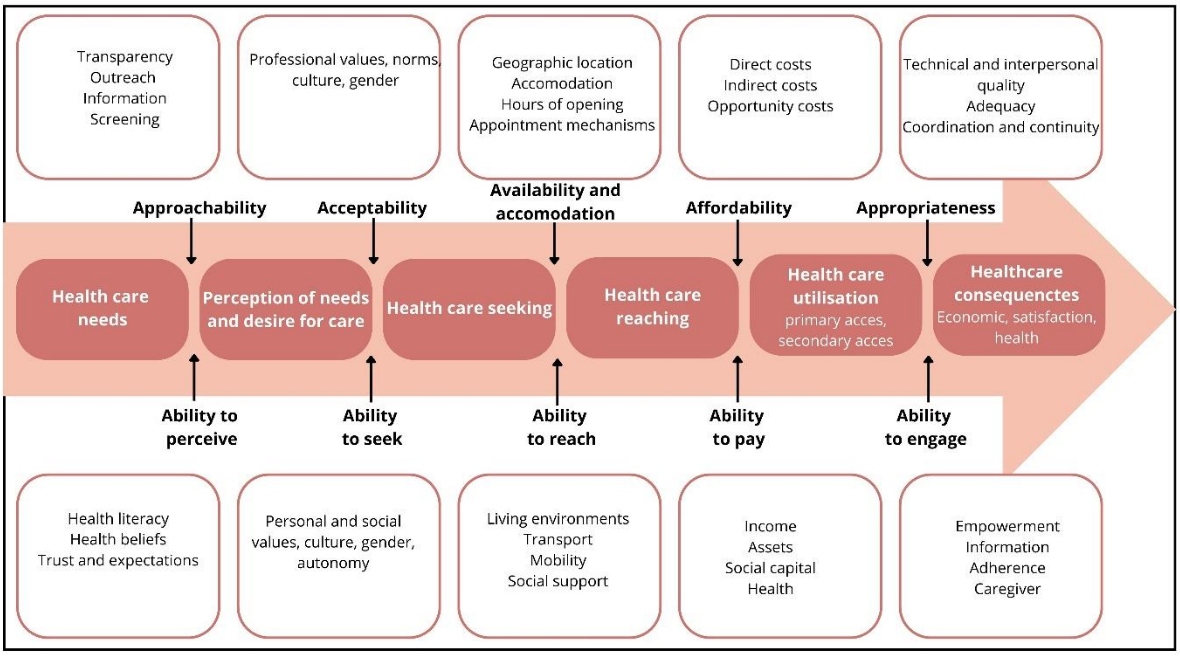

Legend: Conceptual framework of "Access to healthcare" as adopted from Levesque et al [25]. Access reflects the opportunity to identify healthcare needs, seek, reach, obtain, and use services. Five dimensions of accessibility (approachability, acceptability, availability/accommodation, affordability, and appropriateness) interact with corresponding population abilities (ability to perceive, seek, reach, pay, and engage) to generate access. Original framework licensed under CC BY 2.0.

Supplementary material 3: Participant information and sociodemographic data form

Dutch (original version)

Ziek in Beeld interview met ouders

Beste ouder/verzorgende,

Uw kind ligt opgenomen op de afdeling kindergeneeskunde in het Juliana Kinderziekenhuis en wij zouden u graag willen interviewen om de dagen voor opname met u te bespreken. Ons doel is om te achterhalen wat voor u de belangrijkste redenen zijn geweest om contact op te nemen met een arts in de dagen dat uw kind ziek thuis was. Dit interview draagt bij aan het verder ontwikkelen van de vragenlijst 'Ziek in Beeld': het verbeteren en op maat maken van adviezen voor ouders met een kind met koorts na ontslag. Dit interview zal circa 30 tot 45 minuten in beslag nemen en op de kamer van uw kind plaatsvinden. Als u het goed vindt kom ik vandaag graag even bij u langs om hier meer informatie over te geven.

Groet,

Maarten Willemsen – master student geneeskunde

<u>Formulier sociaal demografische gegevens:</u> gegevens voorafgaand aan interview

**Geslacht**	□ Man<br>□ Vrouw<br>□ Anders
**Relatie tot opgenomen kind**	□ Vader<br>□ Moeder<br>□ Anders, namelijk: ……………………………………
**Leeftijd**	…….. jaar
**Woonplaats**	………………………………………………………………
**Gezinssituatie**	□ Getrouwd, geregistreerd partnerschap of samenwonend met partner<br>□ Alleenstaand<br>□ Weduwe/weduwnaar<br>□ Anders, namelijk
**Beroep**	………………………………………………………………
**Aantal kinderen**	………………………………………………………………
**Leeftijd kinderen**	………………………………………………………………
**Leeftijd opgenomen kind**	………………………………………………………………
**Datum opname kind**	………………………………………………………………
**Datum ontslag kind (indien al ontslagen)**	………………………………………………………………

English (translated version)

Ziek in Beeld ("Illness visualized") interview with parents

Dear parent/caregiver,

Your child has been admitted to the paediatric ward at Juliana Children's Hospital, and we would like to interview you to discuss the days leading up to admission. Our aim is to understand what the most important reasons were for you to contact a physician during the days your child was ill at home.

This interview will contribute to the further development of the "Ziek in Beeld" questionnaire, with the aim of improving and tailoring advice for parents of children with fever after discharge. The interview will take approximately 30 to 45 minutes and will take place in your child's room.

If you agree, I would be happy to visit you today to provide more information.

Kind regards,

Maarten Willemsen
Master's student in Medicine

Sociodemographic data form: pre-interview information

**Sex**	□ Male<br>□ Female<br>□ Other
**Relationship to admitted child**	□ Father<br>□ Mother<br>□ Other, namely: ……………………………………
**Age**	…….. jaar
**Place of residence**	…………………………………………………………
**Family situation**	□ Married, registered partnership, or cohabiting with partner<br>□ Single<br>□ Widow/widower<br>□ Other, namely
**Occupation**	…………………………………………………………
**Number of children**	…………………………………………………………
**Age of children**	…………………………………………………………
**Age of admitted child**	…………………………………………………………
**Date of child's admission**	…………………………………………………………
**Date of child's discharge (if already discharged)**	…………………………………………………………

Supplementary material 4: Topic guide

Dutch (original version)

Vragenlijst semigestructureerde interviews voor Ziek in Beeld

1. **Introductie van interview aan participant**

Nogmaals bedankt voor het willen deelnemen aan dit onderzoek. Mijn naam is Maarten Willemsen en ik ben een master student geneeskunde aan de Universiteit van Leiden. Ik doe dit onderzoek in het kader van de wetenschapsstage binnen mijn master. Dit onderzoek valt binnen een groter onderzoek genaamd 'Ziek in beeld'. Dit onderzoek heeft het doel om ouders te helpen een goede inschatting te kunnen maken over hoe ziek uw kind is en of uw kind opnieuw moet worden beoordeeld door een arts. Uw kind is helaas ook ziek geworden. Ik wil in mijn interview leren wanneer u bezorgd werd over uw kind en hoe u besloten heeft om een arts te bellen. De Ziek in Beeld vragenlijst die gebruikt wordt heb ik bij me en zullen wij samen tijdens het beantwoorden van de vragen doornemen.

Ik wil graag samen door het informatieformulier gaan waar de inhoud van het onderzoek en het doel hiervan nog eens beknopt wordt beschreven. Het onderzoek is op vrijwillige basis en informatie die u mij geeft wordt geanonimiseerd, dat wil zeggen dat datgene wat u mij vertelt niet naar u terug te leiden zal zijn en uw naam nergens wordt opgeschreven. De informatie zal daarna alleen door mij, Evelien van Kempen (arts-onderzoeker) en Mirjam van Veen (kinderarts en hoofdonderzoeker van 'Ziek in beeld') te zien zijn. Hier is het informatieformulier. Als u vragen heeft mag u die op elk moment stellen. Wanneer u akkoord bent kunt u het informed consent formulier ondertekenen.

Ik wil u nogmaals benadrukken dat het een vrijwillig interview is en dat we op elk moment dat u dat wilt kunnen pauzeren of stoppen. Als u een vraag niet wilt beantwoorden kunnen we die overslaan. Ik zal van het interview een geluidsopname maken via teams op de computer zodat ik na dit interview uw antwoorden kan bestuderen. Bent u akkoord met het opnemen? Ik zal na het afronden van dit onderzoek in juni 2024 deze opnames verwijderen.

Heeft u nog vragen voordat we het interview gaan beginnen?

2. **Opening**

- Kunt u mij in uw eigen woorden beschrijven wat er met uw kind aan de hand is?

3. **Onderwerpen**

3.1 Factoren die verloop ziektebeeld en besluit tot contact met arts bepaalden

We willen met u de Ziek in Beeld vragenlijst terug in de tijd invullen. Zo willen we kijken welke kenmerken in de loop van de dagen veranderen. We gaan terug naar de dag dat uw kind werd opgenomen in het ziekenhuis. We hebben hiervoor op papier de 'Ziek in Beeld' vragenlijst liggen.

1. Hoe zou u op de dag van opname de vragenlijst hebben ingevuld?
2. Wat waren voor u de belangrijkste kenmerken, die in de 'Ziek in beeld' vragenlijst worden genoemd, om contact op te nemen met een arts?

- Wat was voor u de reden dat deze kenmerken zo belangrijk waren?

We gaan terug naar één dag voordat uw kind werd opgenomen in het ziekenhuis. Als u foto's heeft van uw kind op deze dag houden we die er graag bij om mede daarmee de volgende vragen te beantwoorden.

3. Hoe zou u op deze dag de vragenlijst hebben ingevuld?
- Wat waren voor u belangrijke verschillen in kenmerken, die in de ‘Ziek in beeld’ vragenlijst worden genoemd t.o.v. de dag daarna?
- Hoe veranderde de items/kenmerken?
4. Heeft u die dag contact gehad met een arts?
- Zo ja, wat waren voor u de belangrijkste kenmerken, die in de ‘Ziek in beeld’ vragenlijst worden genoemd, om contact op te nemen met een arts?

Indien van toepassing gaan we nog één dag terug naar twee dagen voordat uw kind werd opgenomen in het ziekenhuis. Als u foto’s heeft van uw kind op deze dag houden we die er graag bij om mede daarmee de volgende vragen te beantwoorden.

5. Hoe zou u op deze dag de vragenlijst hebben ingevuld?
- Wat waren voor u belangrijke verschillen in kenmerken, die in de ‘Ziek in beeld’ vragenlijst worden genoemd t.o.v. de dag daarna?
- Hoe veranderde de items/kenmerken?
6. Heeft u die dag contact gehad met een arts?
- Zo ja, wat waren voor u de belangrijkste kenmerken, die in de ‘Ziek in beeld’ vragenlijst worden genoemd, om contact op te nemen met een arts?

De komende vragen betreffen de gehele periode van ziek zijn van uw kind

7. Welke kenmerken zag u in de loop van het ziektebeeld het meest veranderen?
8. Zijn er kenmerken die in de vragenlijst staan die u niet meeneemt in de beoordeling hoe ziek uw kind is?
9. Zijn er kenmerken die niet in vragenlijst staan die u wel meeneemt in de beoordeling hoe ziek uw kind is?

4. Factoren buiten ziekte-ernst van kind om die besluit tot contact met arts bepaalden

De volgende vragen gaan verder in op uw beslissing contact op te nemen met een arts

10. Heeft u in de dagen dat uw kind ziek was (eerder) contact gehad met een dokter, zoals bijv. de huisarts?
- Wanneer was dat?
- Kunt u vertellen waarom u contact had met de dokter? Wat maakte dat u contact zocht met een dokter?
  a. Heeft u hier op een bepaalde manier informatie over opgezocht? En zo ja, via welke manier (bijvoorbeeld thuisarts, andere website, omgeving)
- Hoe verliep dit?
  a. Met wie had u contact?
  b. Hoe werd uitgelegd wat uw kind had? Was deze uitleg duidelijk voor u?
  c. Voelde u zich gehoord?
  d. Heeft u negatieve ervaringen aan het contact met de arts(en)? Zo ja, kunt u benoemen welke ervaringen?
  e. Heeft u positieve ervaringen aan het contact met de arts(en)? Zo ja, kunt u benoemen welke ervaringen?
  f. Wist u wanneer u opnieuw hulp van een arts moest vragen? Hoe kreeg u hier informatie over en hoe werd dit uitgelegd?

11. Welke verschillende mogelijkheden om contact op te nemen met een arts kent u?
12. Heeft uw gedachten over bepaalde artsen uw keuze veranderd om met deze arts contact op te nemen?

- Zo ja, welke meningen zijn dit?

13. Heeft de plek waar het ziekenhuis staat, de afstand tot uw huis of de bereikbaarheid tot de arts uw keuze veranderd om contact op te nemen met een arts?

- Zo ja, welk van bovengenoemde dingen heeft uw keuze veranderd en waarom?

14. Hebben eventuele kosten uw keuze veranderd om contact op te nemen met een arts?

- Zo ja, kunt u dit toelichten?

15. Zijn er voor u bepaalde redenen waarom u twijfelde om contact op te nemen met een bepaalde arts?

- Zo ja, welke zijn dit?

16. Zijn er andere redenen geweest, buiten het ziek zijn van uw kind om, die u sneller óf minder snel contact hebben laten opnemen met een arts?

- Welke redenen zijn dit?

Slot:

17. Heeft u nog andere opmerkingen die u graag met ons wilt delen die nu niet benoemd zijn naar aanleiding van de ziekteperiode van uw kind?

Bedankt voor uw medewerking. We wensen u veel sterkte de komende periode en hopen dat uw kind snel hersteld.

Stelt u verdere informatie over de uitkomst van het onderzoek op prijs? U kunt hier uw emailadres invullen en dan zullen wij dat na afronding van mijn stage naar u toesturen.

- Duur interview:

English (translated version)

<u>Topic guide for semi-structured interview Ziek in Beeld</u>

1. Introduction of the interview to the participant

Thank you again for agreeing to participate in this study. My name is Maarten Willemsen, and I am a medical master's student at Leiden University. I am conducting this study as part of my research internship within the master's program. This study is part of a larger project called Ziek in Beeld ("Illness Visualized"). The aim of this research is to help parents make an accurate assessment of how ill their child is and whether the child needs to be reassessed by a doctor. Unfortunately, your child also became ill.In this interview, I would like to learn when you became concerned about your child and how you decided to contact a doctor. I have the Ziek in Beeld questionnaire with me, and we will go through it together while answering the questions.

I would like to briefly go through the information sheet with you, which summarizes the content and purpose of the study. Participation is voluntary, and any information you provide will be anonymized, meaning that what you tell me cannot be traced back to you, and your name will not be recorded. The information will only be accessible to me, Evelien van Kempen (physician-researcher), and Mirjam van Veen (pediatrician and co-principal investigator of Ziek in Beeld). Here is the information sheet. You may ask questions at any time. If you agree, you may sign the informed consent form.

I want to emphasize again that this interview is voluntary, and we can pause or stop at any time. If you do not want to answer a question, we can skip it. I will record the interview via Teams so I can study your answers afterward. Do you agree to the recording? I will delete these recordings after completing the study in June 2024.

Do you have any questions before we start the interview?

2. Opening question

- Could you describe in your own words what is going on with your child?

3. Topics

3.1 Factors determining illness progression and the decision to contact a doctor

We want to fill in the Ziek in Beeld questionnaire with you retrospectively to see which features changed over the course of the days. We will go back to the day your child was admitted to the hospital. We have the questionnaire on paper.

1. How would you have completed the questionnaire on the day of admission?
2. Which features listed in the *Ziek in Beeld* questionnaire were most important to you in deciding to contact a doctor?
    - Why were these features so important to you?

We will now go back to one day before your child was admitted. If you have photos of your child on that day, we can use them to help answer the following questions.

3. How would you have completed the questionnaire on that day?

- What important differences in features did you notice compared with the following day?
- How did the items/features change?
4. Did you have contact with a doctor on that day?
- If yes, which features listed in the questionnaire prompted you to contact a doctor?

If applicable, we will go back to two days before admission. Again, photos may help answer the next questions.

5. How would you have completed the questionnaire on that day?
- What important differences in features did you notice compared with the following day?
- How did the items/features change?
6. Did you have contact with a doctor on that day?
- If yes, which features listed in the questionnaire prompted you to contact a doctor?

The following questions relate to the entire period during which your child was ill:

7. Which features did you notice changing most over the course of your child's illness?
8. Are there features listed in the questionnaire that you did not consider when assessing how sick your child was?
9. Are there features not listed in the questionnaire that you did consider when assessing your child's illness?

4. Factors beyond the child's illness severity affecting the decision to contact a doctor

The next questions focus on your decision to contact a doctor:

10. During the days your child was ill, did you contact a doctor (e.g., your general practitioner)?
- When was this?
- Can you explain why you contacted the doctor? What prompted you to seek help?
  a. Did you look up any information beforehand? If so, how (e.g., Thuisarts, other website, social circle)?
- How did the consultation go?
  a. Who did you speak with?
  b. How was your child's condition explained? Was it clear?
  c. Did you feel heard?
  d. Did you have any negative experiences with the doctor(s)? If yes, please specify.
  e. Did you have any positive experiences with the doctor(s)? If yes, please specify.
  f. Did you know when to seek help again? How was this information communicated?

11. What different ways of contacting a doctor do you know?
12. Did your opinions about certain doctors influence your choice to contact a specific doctor?
- If yes, which opinions?
13. Did the hospital's location, distance from your home, or accessibility influence your choice to seek care?
- If yes, which factors affected your decision and why?
14. Did any costs influence your decision to contact a doctor?
- If yes, please explain.

15. Were there reasons you hesitated to contact a particular doctor?
- If yes, what were these reasons?
16. Were there other factors, apart from your child's illness, that made you contact a doctor sooner or later?
- What were these factors?

Closing questions

17. Do you have any other comments about your child's illness period that you would like to share?

Thank you for your participation. We wish you strength in the coming period and hope your child recovers quickly.

Would you like to receive further information about the study's results? You can provide your email, and we will send it to you after the completion of my internship.

Duration of interview:

Supplementary material 5: Table S1: overview themes with illustrative quotes

Theme	Subtheme	Illustrative quote(s)
1. In parents' perspective abnormal illness of the child	1.1 Severe factor(s) prompting immediate care	"After his nap, I almost couldn't wake him up and thought it might be serious enough to call an ambulance" (P3); "He would lose consciousness because he was simply exhausted. It seemed every time like he was about to faint and would not wake up again" (P9); ʹHe would lose consciousness every time, but when he was awake, he didn't fully realize it. He would be eating a cracker that would fly out of his mouth and then ask why the wall is pink"(P15); "She was completely cold, turned extremely pale, and had blue lips………I thought she was going to die" (P4); "She had spots on her cheeks, which is a rash indicative of sepsis"(P8); "His chest contracted a little and his breathing became heavier" (P11); "She had a high fever and was crying non-stop. She was very restless and not herself…."(P5); "She was squealing… overstretching… not in a good mood"(P4)
	1.2 Combination of non-severe factors	"A fever in itself is not that bad… but because it was a combination of factors we contacted the GP" (P6)
2. Recognition of symptoms from the past, based on the medical history of patient and family	2.1 Child's prior (medical) history	"Retractions in her neck and ribs, the abdomen having to work harder, and nasal flaring are all things we know to watch for." (P13); "A year ago, he had the RSV virus for which he was hospitalized. We initially thought that was the case again and therefore we contacted the GP" (P1); "My daughter previously had meningitis. Now I saw her not playing again, with a high fever, red cheeks, pallor, vomiting, and chills. I recognized these symptoms from earlier…..." (P8); "He has been seriously ill multiple times due to pyelonephritis and has frequently been hospitalized. If his temperature goes above 38.5 degrees Celsius, I always call (the doctor); "With her, it is very typical for things to escalate quickly."(P4)
	2.2 Previous parental illness with similar symptoms	"My wife had dengue… we immediately thought: could this be the same thing?" (P5)
3. In parents' perception an excessively long illness duration		"Fever usually peaks after a few days, but this time it persisted, so there was something wrong" (P3); "She was having difficulty breathing… it lasted longer than 3 days, so I thought, let's go to the doctor" (P7)
4. Delay in seeking medical care due to parental work commitments		"I had a shift to work on Monday, otherwise I think I would have already gone to the GP on Monday, it ended up being Tuesday… it was actually only getting worse" (P3); "Without my work concerns, I would have sought contact earlier" (P5)

5. Absence of own general practitioner, leading to earlier or delayed medical consultation		"It was Easter weekend… On Tuesday, the GP who had previously seen him, was working, so we thought it would be most practical to consult him then. (P2); "She had trouble in the morning, so we thought it was best to go immediately… otherwise, she would have to go to the after-hours clinic" (P6)
