# Supplementary material for: Understanding parents’ decisions to seek care for children with serious bacterial infections: a qualitative study
Source: BMJ Open. 2026 Jun 24;16(6):e115270. doi: 10.1136/bmjopen-2025-115270 (PMC13296029; doi:10.1136/bmjopen-2025-115270)
Supplement: Supplementary file 2 [file bmjopen-2025-115270.reviewer_comments.pdf]

PEER REVIEW HISTORY

BMJ Open publishes all reviews undertaken for accepted manuscripts. Reviewers are asked to complete a checklist review form and are provided with free text boxes to elaborate on their assessment. These free text comments are reproduced below.

ARTICLE DETAILS

Title (Provisional)

UNDERSTANDING PARENTS' DECISIONS TO SEEK CARE FOR CHILDREN WITH SERIOUS BACTERIAL INFECTIONS: A QUALITATIVE STUDY

Authors

van Kempen, Evelien; Willemsen, Maarten; Oostenbrink, Rianne; van Veen, Mirjam

---

VERSION 1 - REVIEW

**Reviewer**	**1**
**Name**	**Green, Kile**
**Affiliation**	**Newcastle University, NIHR In Vitro Diagnostics Co-operative**
**Date**	**12-Jan-2026**
**COI**	**None**

Thank you for the opportunity to review this article. This appears to be a short qualitative study to determine factors influencing help-seeking for parents of young children experiencing suspected bacterial infection. Overall the content is well-written and clear, with some interesting insights.

The introduction sets the scene well, and defines the purpose of the article within the wider context of the study. There is perhaps some blending of methods into the latter section of the introduction, but this could be considered setting-the-scene.

Methodology and frameworks seem appropriate and well described. While I have some mild concerns regarding the Kuijper's questionnaire itself, I'm not familiar with it and the questionnaire itself is outside of the scope of this qualitative publication.

Study conduct appears appropriate, as do participation criteria.

I noticed a requirement for Dutch language proficiency, but quotes are presented in English. It isn't clear how they were translated. Please do note this in the methods if they were translated, and consider how the translation may invite interpretation bias in limitations.

Apologies if I have misunderstood, but the other aspect that was unclear was the intersection between the e-health questionnaire and the qualitative interviews - I expect the

study populations for the tool and the interviews were entirely separate, but it is unclear. Use of the tool would likely bias participant responses.

A little more information on the PPIE activities and number of PPIE contributors would be a nice addition, but not essential to the overall article.

Ethics appear appropriate.

Thank you for supplying the associated supplementary materials including the completed COREQ checklist and topic guide with an English translation, that has been very helpful. The layout and format of the interviews is very similar to the style we use at the NIHR HRC so it was easy to follow along and understand your process.

Including a copy of any other documents used in this study would be helpful, particularly copies of participant information sheets and consent templates. You could consider (if appropriate and consented to) including the anonymised transcripts to allow secondary analysis in the interest of open research practices, but understand this may not be feasible at this stage.

Results are generally well-described, with some minor typo's causing some initial confusion (article pg 9 ln 52-58). It may be helpful for the reader if you reiterate the aims noted in this section as I had to scroll back to find them. Otherwise the results are well-described and succinct, with a suitable structure.

Discussion is clear and aligns with the results, adequately supported with comparison to similar studies. It is unclear how many participants had prior experience of serious illness, but this is noted as an important factor in the discussion and may explain differences in perceived awareness of clinical symptoms in this cohort compared to others (pg 16, lines 32-44). There are perhaps other demographic characteristics that may have influenced results, which are touched upon in the implications section. Any more contextual information you could provide on the cohort that may have influenced your findings would be good to note.

Thank you again for preparing this article, it was an interesting read. A few minor clarifications will improve understandability in an otherwise well-conducted qualitative study.

**Reviewer**	**2**
**Name**	**Dunne, Colum**
**Affiliation**	**University of Limerick, Centre for Interventions in Infection**
**Date**	**13-May-2026**
**COI**	**None**

I have read this manuscript carefully and with interest. The authors have completed a useful study of parents' information seeking, following illness of their young children, detailing circumstances of the parents and diagnosis of the children.

The manuscript is well-prepared, well-written, with clearly presented findings.

The study design, based on survey, is not complicated and the findings are not overly elaborated.

I have one recommendation, which relates only to the scale of the work and the sample size. I would like the authors to include two expanded elements: specifically, to detail the scale of other comparable studies and therefore allow a reader decide easily whether the findings here are generalisable based on scale of other published works, and include reference to other works beyond Kuijpers which I believe has been overly relied on.

These comments are intended to be helpful

VERSION 1 - AUTHOR RESPONSE

Reviewer 1:

Thank you for the opportunity to review this article. This appears to be a short qualitative study to determine factors influencing help-seeking for parents of young children experiencing suspected bacterial infection. Overall the content is well-written and clear, with some interesting insights.
The introduction sets the scene well, and defines the purpose of the article within the wider context of the study.

Comment 1: There is perhaps some blending of methods into the latter section of the introduction, but this could be considered setting-the-scene.
Answer: Thank you for comment. It was indeed intended to set the scene. We have retained this text, while ensuring that detailed methodological information is presented in the Methods section.

Methodology and frameworks seem appropriate and well described. While I have some mild concerns regarding the Kuijper's questionnaire itself, I'm not familiar with it and the questionnaire itself is outside of the scope of this qualitative publication.

Study conduct appears appropriate, as do participation criteria.

Comment 2: I noticed a requirement for Dutch language proficiency, but quotes are presented in English. It isn't clear how they were translated. Please do note this in the methods if they were translated, and consider how the translation may invite interpretation bias in limitations.
Answer: Thank you for noting this. The interviews were conducted and transcribed in Dutch, and illustrative quotes were translated into English for publication. We have clarified this in the Methods section. We have also added translation as a potential source of interpretation bias in the Limitations section.

Page 7, line 152-153
The interviews were transcribed ad verbatim in Dutch by MW, including non-verbal information.

Page 7, line 161-162
For publication, selected illustrative quotes were translated into English by the research team, with attention to preserving participants' intended meaning.

Page 18, line 431--434
Fourth, as interviews were conducted in Dutch and quotes were translated into English for publication, some nuance may have been affected during translation, although translations were checked by the research team to preserve participants' intended meaning.

Comment 3: Apologies if I have misunderstood, but the other aspect that was unclear was the intersection between the e-health questionnaire and the qualitative interviews - I expect the study populations for the tool and the interviews were entirely separate, but it is unclear. Use of the tool would likely bias participant responses.
Answer: Thank you for comment. Please see also our response to "editor comment 3" regarding the position of the e-health tool in relation to the present study. Parents in the present study did not use the e-health tool; the structured questionnaire underlying the tool was used as a reference and formed part of the topic guide to support discussion of illness characteristics and to explore whether the included signs and symptoms aligned with parents' experiences of serious bacterial infection. We have clarified this in the Methods section.

Page 7, line 165-166
The parents participating in the present qualitative study were separate from those involved in this earlier work and did not use the e-health tool during the study.

Comment 4: A little more information on the PPIE activities and number of PPIE contributors would be a nice addition, but not essential to the overall article.
Answer: We thank the reviewer for this helpful suggestion. We have added brief information on the PPIE activities. We kept this description concise to avoid shifting the focus of the present article toward the detailed development of the e-health tool; further details on the questionnaire development are described by Kuijpers et al.

Page 7, line 163-169
Parents were actively involved in the development of the questionnaire and e-health tool currently under development. The parents participating in the present qualitative study were separate from those involved in this earlier work and did not use the e-health tool during the study. The present study further informed the tool and its underlying questionnaire by exploring parents' experiences of recognising signs of a SBI and seeking medical care. Parents were not involved in the recruitment, conduct, analysis, or dissemination plans of this study.

Ethics appear appropriate.

Thank you for supplying the associated supplementary materials including the completed COREQ checklist and topic guide with an English translation, that has been very helpful. The layout and format of the interviews is very similar to the style we use at the NIHR HRC so it was easy to follow along and understand your process.

Comment 5: Including a copy of any other documents used in this study would be helpful, particularly copies of participant information sheets and consent templates. You could consider (if appropriate and consented to) including the anonymised transcripts to allow secondary analysis in the interest of open research practices, but understand this may not be feasible at this stage.

Answer: Thank you for comment. We have included the participant information sheet and consent form as supplementary materials. While we acknowledge the value of sharing anonymised qualitative data for secondary analysis, we are unfortunately unable to make the interview transcripts available at this stage. In addition, the interviews were conducted and transcribed in Dutch, which would further limit their direct usability for an international readership.

Supplementary Material 3
Informed consent form (separate as requested by the Editorial Office)

Comment 6: Results are generally well-described, with some minor typo's causing some initial confusion (article pg 9 ln 52-58).
Answer: We thank the reviewer for noting this. Although the line numbers mentioned by the reviewer did not correspond to page 9 in our manuscript version, we reviewed the Results section thoroughly and corrected some minor typo's.

Comment 7: It may be helpful for the reader if you reiterate the aims noted in this section as I had to scroll back to find them. Otherwise the results are well-described and succinct, with a suitable structure.
Answer: Thank you for your suggestion. We have added a brief orienting sentence at the start of the Results section to reiterate the study aims.

Page 10, line 205-207
The first three themes related to the primary aim and reflected the factors parents perceived as most distinctive in prompting them to seek medical care. This is captured by the overarching theme of perceived abnormal illness.

Page 10, line 217-219
Two themes were identified in relation to the secondary aim, reflecting factors beyond the child's health characteristics that parents experienced as facilitating or hindering their decision to seek medical care.

Discussion is clear and aligns with the results, adequately supported with comparison to similar studies.

Comment 8: It is unclear how many participants had prior experience of serious illness, but this is noted as an important factor in the discussion and may explain differences in perceived awareness of clinical symptoms in this cohort compared to others (pg 16, lines 32-44).
Answer: We agree with your comment. Unfortunately, we did not systematically collect this information as a separate participant characteristic. Prior experience emerged inductively during the interviews when parents spontaneously described previous encounters with serious illness or hospital care. We have clarified this in the discussion.

Page 16, line 379-381
Our findings suggest that parents, especially those who mentioned previous experience, are (more) aware of clinical warning signs of serious illness, surpassing the recognition observed in earlier studies [9,21,22,28].

Page 18, line 422-419
Secondly, the sample included several parents with previous experience navigating (in-)hospital care, which allowed us to identify signs they considered distinctive for SBI. However, prior experience was not systematically collected as a participant characteristic, so we could not determine how many parents had such experience. This may limit transferability to parents with less experience of navigating healthcare and should be considered when interpreting the role of prior experience in symptom awareness.

Comment 9: There are perhaps other demographic characteristics that may have influenced results, which are touched upon in the implications section. Any more contextual information you could provide on the cohort that may have influenced your findings would be good to note.

Answer: Thank you for this suggestion. We have added a reflection to the implications section noting that healthcare-related occupations, previous experience with serious illness or hospital care and parental role may have shaped parents' symptom recognition, description of severity, and care-seeking decisions. Because of the small sample and need to protect anonymity, we describe these characteristics in general terms rather than at participant level.

Page 19, line 448-453
These findings should be interpreted in light of the characteristics of the participating parents. Several parents had healthcare-related occupations or previous experience with serious illness or hospital care, which may have influenced their familiarity with alarming signs, their ability to describe symptoms, and their threshold for seeking medical care. Most participants were mothers, although fathers also participated, and differences in caregiving roles may have influenced which illness signs were observed and emphasised during the interviews.

Thank you again for preparing this article, it was an interesting read. A few minor clarifications will improve understandability in an otherwise well-conducted qualitative study.

Reviewer 2:

I have read this manuscript carefully and with interest. The authors have completed a useful study of parents' information seeking, following illness of their young children, detailing circumstances of the parents and diagnosis of the children.
The manuscript is well-prepared, well-written, with clearly presented findings.
The study design, based on survey, is not complicated and the findings are not overly elaborated.

I have one recommendation, which relates only to the scale of the work and the sample size.

Comment 1: I would like the authors to include two expanded elements: specifically, to detail the scale of other comparable studies and therefore allow a reader decide easily whether the findings here are generalisable based on scale of other published works,

Answer: Thank you for this comment. We have expanded the Discussion to include the sample sizes of the two comparable qualitative studies, Bruel et al. and Sahm et al., to help readers interpret the scale of our study in relation to previous work.

Page 16, line 381-387
Our findings align with previous qualitative studies exploring parental recognition of serious illness and childhood fever. Bruel et al., in a study of 18 children hospitalised with a serious infection, found that parents identified drowsiness, irritability and different crying as key signs, along with their impression that illness was different [6]. Similarly, Sahm et al. conducted semi-structured interviews with 23 parents of children aged ≤5 years and found that petechiae and neck stiffness prompted parents to seek emergency care [13].

Comment 2: and include reference to other works beyond Kuijpers which I believe has been overly relied on.
Answer: We thank the reviewer for this helpful comment.We have revised the Introduction to clarify that similar findings have been reported by other studies and added additional references in the Discussion.

Page 4, line 86-88
Parents may find it difficult to recognise specific clinical symptoms, like dyspnoea or dehydration, which are commonly used in physician instructions, especially when they had no prior experience with these symptoms. [9, 21,22]

Page 5, line 91-92
Building on this work, we initiated a phased project with the ultimate aim of developing an e-health tool to support parents in assessing the severity of febrile illness in young children.

Page 16, line 374-375
Based previous research, we anticipated that parents would focus more on general behavioral changes than on specific clinical symptoms [9,21,22].

These comments are intended to be helpful

VERSION 2 - REVIEW

**Reviewer**	**1**
**Name**	**Green, Kile**
**Affiliation**	**Newcastle University, NIHR In Vitro Diagnostics Co-operative**
**Date**	**26-May-2026**
**COI**	

I thank the authors for their effort to revise this document and provide supporting content where it was possible to do so. I am satisfied with their changes and feel this article is now

suitable for publication following editorial review. I have no additional comments or concerns of note. Congratulations on the completion of this interesting and original work.
